# *Bauhinia championii* Flavone Attenuates Hypoxia-Reoxygenation Induced Apoptosis in H9c2 Cardiomyocytes by Improving Mitochondrial Dysfunction

**DOI:** 10.3390/molecules21111469

**Published:** 2016-11-04

**Authors:** Ping Liao, Guibo Sun, Chan Zhang, Min Wang, Yao Sun, Yuehan Zhou, Xiaobo Sun, Jie Jian

**Affiliations:** 1 Department of Pharmacology, Guilin Medical University, Huan Cheng North 2nd Road, Guilin 541004, Guangxi, China; liaoping555@163.com (P.L.); 15807731320@163.com (C.Z.); sandysun318@163.com (Y.S.); yuehanzhou2012@163.com (Y.Z.); 2 Institute of Medicinal Plant Development, Peking Union Medical College and Chinese Academy of Medical Sciences, Beijing 100193, China; sunguibo@126.com (G.S.); lily_12506053@163.com (M.W.)

**Keywords:** *Bauhinia championii* flavone, hypoxia-reoxygenation, apoptosis, mitochondrial dysfunction, PI3K/Akt

## Abstract

This study aimed to determine the effects of *Bauhinia championii* flavone (BCF) on hypoxia-reoxygenation (H/R) induced apoptosis in H9c2 cardiomyocytes and to explore potential mechanisms. The H/R model in H9c2 cardiomyocytes was established by 6 h of hypoxia and 12 h of reoxygenation. Cell viability was detected by CCK-8 assay. Apoptotic rate was measured by Annexin V/PI staining. Levels of mitochondria-associated ROS, mitochondrial transmembrane potential (∆Ψm) and mitochondrial permeability transition pores (MPTP) opening were assessed by fluorescent probes. ATP production was measured by ATP assay kit. The release of cytochrome c, translocation of Bax, and related proteins were measured by western blotting. Our results showed that pretreatment with BCF significantly improved cell viability and attenuated the cardiomyocyte apoptosis caused by H/R. Furthermore, BCF increased ATP production and inhibited ROS-generating mitochondria, depolarization of ΔΨm, and MPTP opening. Moreover, BCF pretreatment decreased Bax mitochondrial translocation, cytochrome c release, and activation of caspase-3, as well as increased the expression of p-PI3K, p-Akt, and the ratio of Bcl-2 to Bax. Interestingly, a specific inhibitor of phosphatidylinositol 3-kinase, LY294002, partly reversed the anti-apoptotic effect of BCF. These observations indicated that BCF pretreatment attenuates H/R-induced myocardial apoptosis strength by improving mitochondrial dysfunction via PI3K/Akt signaling pathway.

## 1. Introduction

Coronary artery disease is the largest contributor to cardiovascular diseases which has become a leading cause of death worldwide [1,2]. Although early myocardial reperfusion using either thrombolytic or primary percutaneous coronary intervention can effectively salve the damaged myocardium, the process of reperfusion can itself lead to further injury such as cardiomyocyte death, which is known as myocardial ischemia/reperfusion (I/R) injury [3]. Consequently, to improve clinical effects in acute MI, it is necessary to develop a cardioprotective drug that could alleviate I/R injury.

Multiple studies have demonstrated that apoptosis contributes to one of the important pathological mechanisms of I/R injury, while the mitochondrial dysfunction associated with intrinsic apoptosis is considered crucial in myocardial apoptosis [4,5,6]. On the other hand, the phosphoinositide 3-kinase/serine/threonine protein kinase (PI3K/Akt) pathway, an important antiapoptosis/proliferation signaling pathway, is known to play a pivotal role in regulating the survival and apoptosis of cardiomyocytes [7]. Various studies have demonstrated that phospho-Akt can improve mitochondrial dysfunction by regulating Bcl-2 protein family [8,9,10]. Therefore, it may be a possible target of improving I/R injury to inhibit apoptosis by activating Akt and improving mitochondrial function.

*Bauhinia championii* (Benth.) Benth. a traditional Chinese medicinal herb，is widely distributed in Guangxi Province of China [11]. A host of studies have demonstrated that extracts of *Bauhinia championii* promote blood circulation, remove blood stasis, and possess anti-oxidative, anti-inflammatory, and anti-platelet aggregative effects [11,12]. *Bauhinia championii* flavone (BCF) is the primary active component of the stem extract. Our previous studies have exhibited the protective effect of BCF against myocardial ischemia/reperfusion Injury via the PI3K/Akt pathway in rats [13,14]. However, the effects of BCF on hypoxia/reoxygenation (H/R)-induced cardiomyocyte apoptosis and its molecular mechanism have not been investigated.

Accordingly, the aim of the present research was to explore whether BCF could attenuate H/R-induced cardiomyocytes apoptosis and mitochondrial dysfunction and to further determine the role of the PI3K/Akt pathway in BCF-induced cardioprotection on H/R.

## 2. Results

### 2.1. BCF Mitigated H/R Induced Apoptosis

**Figure 1:**
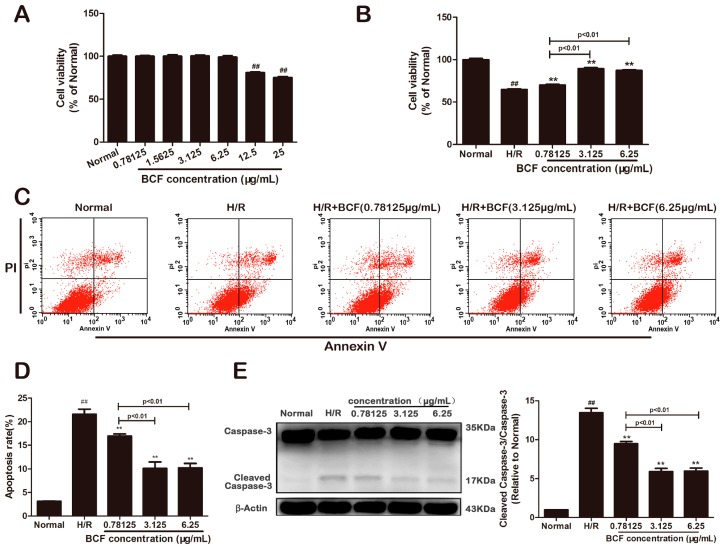
Effects of BCF on H/R induced myocardial apoptosis. H9c2 cells were pretreated with the indicated BCF concentrations for 4 h followed by 6 h of hypoxia and 12 h of reoxygenation or pretreated with indicated BCF concentrations for 24 h before the detection of relevant indexes. (**A**) Cytotoxicity was detected by CCK-8 assay; (**B**) Cell viability was determined by CCK-8 assay; (**C**) The apoptotic ratio of H9c2 cardiomyocytes was measured by flow cytometry using Annexin V-FITC and PI staining; (**D**) The apoptosis rate was quantified by BD FACS software; (**E**) Caspase-3 and cleaved caspase-3 was detected by western blotting. Values were represented as mean ± SD (*n* = 6, each group). ^##^ *p* < 0.01 vs. normal; ** *p* < 0.01 vs. H/R-treated cells.

The cytotoxic test showed that no change in cell viability was found after 24 h of pretreating with various BCF concentrations (*p* < 0.01, Figure 1A). Compared with the normal group, cardiomyocytes subjected to H/R exhibited a significant decrease in viability and increase in apoptosis, while three dosage of BCF protected the H9c2 cardiomyocytes against H/R injury (*p* < 0.01, Figure 1B–D). Besides, the apoptosis marker, cleaved caspase-3 and caspase-3 were detected by western blotting. As shown in Figure 1E, the activation of caspase-3 in the H/R group was higher than that in the normal group (*p* < 0.01). Compared with the H/R group, BCF preconditioning markedly downregulated the activation of caspase-3 (*p* < 0.01). These results suggested that BCF protected the cells against H/R-induced apoptosis and 3.125 μg/mL was the most effective dose. Therefore, a dosage of 3.125 μg/mL was chosen for further experiments.

### 2.2. BCF Pretreatment Activated PI3K/Akt Signaling Pathway

**Figure 2:**
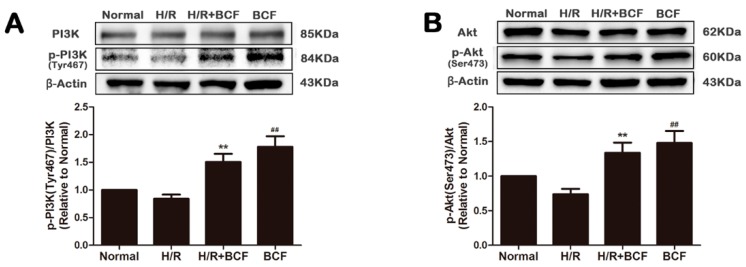
Effect of BCF on PI3K/Akt signaling pathway. Pretreatment with BCF (3.125 μg/mL) for 4 h prior to H/R, the protein expression of PI3K (**A**), p-PI3K (Tyr467) (**A**), Akt (**B**), and p-Akt (Ser473) (**B**) was measured by western blotting. Quantitative analysis of the ratio of p-PI3K (Tyr467) to PI3K and p-Akt (Ser473) to Akt in protein expression were evaluated. Values were represented as mean ± SD (*n* = 6, each group). ** *p* < 0.01 vs. H/R group. ^##^ *p* < 0.01 vs. normal group.

To explore whether the cardioprotection of BCF connected with the modulation of PI3K/Akt pathway, some crucial proteins, such as PI3K and Akt were tested by western blotting. As shown in Figure 2, compared with the H/R group, the expression of p-PI3K (Tyr467) and p-Akt (Ser473) was significantly increased by BCF pretreatment (*p* < 0.01). The results suggested that BCF might activate PI3K/Akt pathway by increasing the phosphorylation of PI3K and Akt.

### 2.3. Inhibition of PI3K Attenuated BCF-Induced Cardioprotection

**Figure 3:**
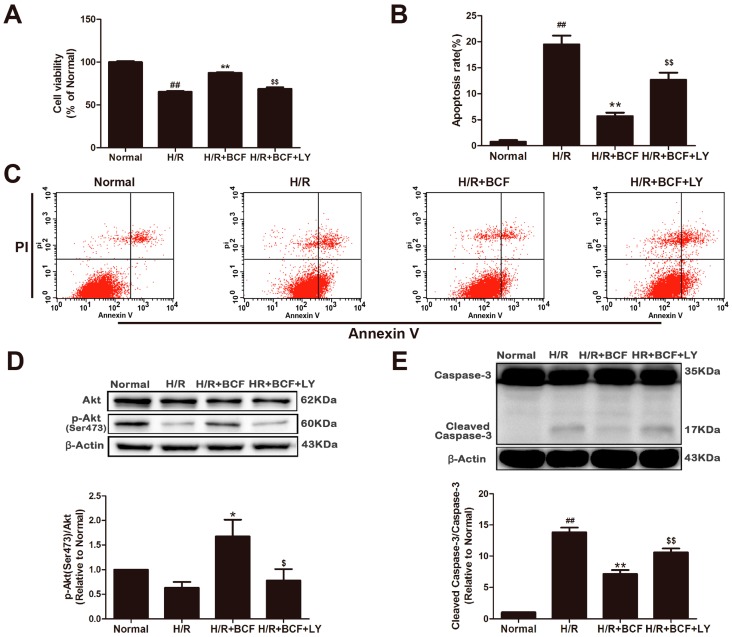
Blocking the PI3K/Akt pathway attenuated BCF-induced cardioprotection. H9c2 cells were pre-incubated with 20 μM LY294002 for 1 h, and then pretreated with BCF (3.125 μg/mL) for 4 h followed by H/R. (**A**) Cell viability was determined by CCK-8 assay; (**B**) The apoptotic ratio of H9c2 cardiomyocytes was measured by flow cytometry using Annexin V-FITC and PI staining; (**C**) The apoptosis rate was quantified by BD FACS software. Effects of BCF and the PI3K inhibitor (LY294002) on p-Akt (Ser473) (**D**); cleaved caspase-3 (**E**) and caspase-3 (**E**) expression levels in H/R-treated cardiomyocytes. β-Actin expression was examined as the protein loading control. Values were represented as mean ± SD (*n* = 6, each group). ^##^ *p* < 0.01 vs. normal; * *p* < 0.05, ** *p* < 0.01 vs. H/R-treated cells; ^$^ *p* <0.05, ^$$^ *p* < 0.01 vs. H/R + BCF-treated cells.

To further investigate if PI3K/Akt activation is essential for the anti-apoptotic effect of BCF, LY294002, a PI3K specific inhibitor was used. As shown in Figure 3, compared with the H/R group, pretreatment with BCF resulted in a marked reduction of cell apoptosis (*p* < 0.01) and the activation of caspase-3, as well as increase of cell viability and p-Akt (Ser473) expression (*p* < 0.01 or *p <* 0.05). However, these effects were partially alleviated after co-administering LY294002 (*p* < 0.01 or *p <* 0.05). Therefore, the PI3K/Akt pathway is involved in the anti-apoptotic effect of induced by BCF.

### 2.4. BCF Inhibited H/R-Induced Mitochondrial Dysfunction via PI3K/Akt Signaling Pathway

**Figure 4:**
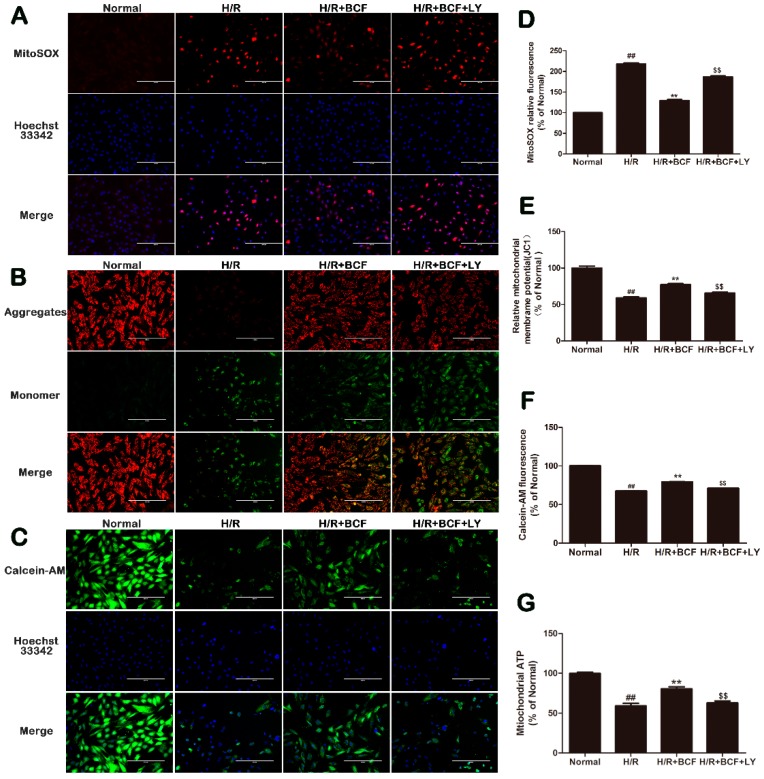
Effects of BCF on ROS-generating mitochondria, ∆Ψm, mitochondrial ATP production, and MPTP opening levels. (**A**) Mitochondria-associated ROS levels were detected by staining cells with MitoSOX (red). Nuclei were stained with blue-fluorescent Hoechst 33342; (**B**) Depolarization of ΔΨm was measured by Fluorescence Microscope using JC-1 probe; (**C**) Changes of MPTP opening in H9c2 cardiomyocytes were detected by calcein-AM (green) as a fluorescence indicator by fluorescence micrographs. Nuclei were stained with blue-fluorescent Hoechst 33342. The scaleplate represents 200 μm. All quantitative analysis of the florescence intensity was detected by a microplate reader. The corresponding histograms (**D**,**E**,**F**) were displayed. (**G**) Mitochondrial ATP production in H9c2 cardiomyocytes was determined using the ATP assay kit. Values were represented as mean ± SD (*n* = 6, each group). ^##^ *p* < 0.01 vs. normal; ** *p* < 0.01 vs. H/R-treated cells; ^$$^ *p* < 0.01 vs. H/R + BCF-treated cells.

It is well known that mitochondria play an important role during hypoxia/reoxygenation-induced apoptosis [4]. In our study, the levels of mitochondria-associated ROS, ∆Ψm, MPTP opening, and mitochondrial ATP production were detected to evaluate the effects of BCF on mitochondrial dysfunction. As shown in Figure 4A,D compared with the H/R group, ROS-generating mitochondria significantly decreased in the H/R + BCF group (*p* < 0.01), but this effect was partly suppressed by LY294002 (*p* < 0.01). Mitochondrial depolarization was indicated by a decrease in the red/green fluorescence intensity ratio of JC-1 staining (*p* < 0.01, Figure 4B,E). The H/R group exhibited an increase in green fluorescence intensity, which indicated ∆Ψm dissipation, while the H/R + BCF group attenuated ∆Ψm dissipation (*p* < 0.01). Compared with the H/R + BCF group, the H/R + BCF + LY group aggravated the loss of ∆Ψm (*p* < 0.01). As the results showed in Figure 4C,F, the H/R group displayed calcein fluorescence loss, which indicated that the MPTP was opening. Interestingly, pretreatment with BCF reduced the MPTP opening induced by H/R but this effect was partially inhibited after co-administering with LY294002 (*p* < 0.01). In addition, mitochondrial ATP production in cardiomyocytes was reduced in the H/R group, while BCF pretreatment increased ATP production (*p* < 0.01, Figure 4G). However, the protective effect of BCF was partially inhibited by co-administering with LY294002.

**Figure 5:**
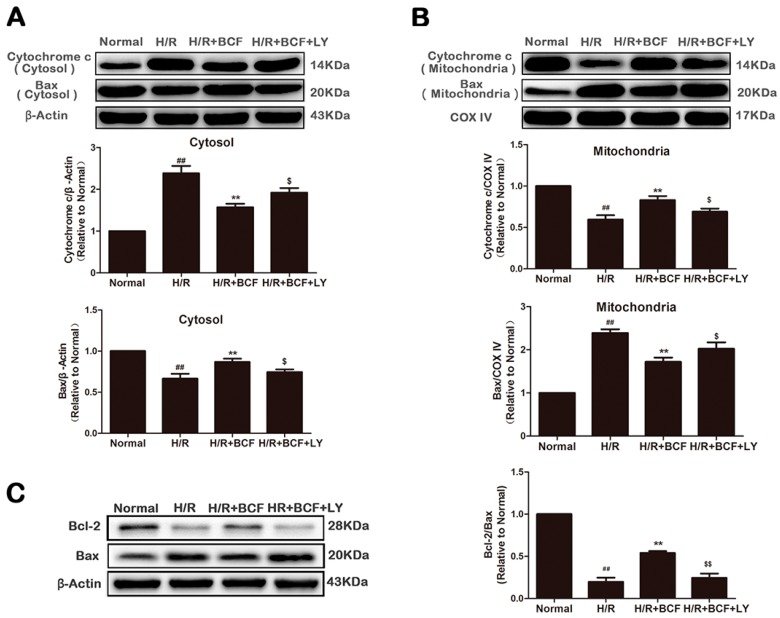
Effects of BCF pretreatment on Bax translocation, release of cytochrome c and the ratio of Bcl-2 to Bax in H/R-treated H9c2 cells. (**A**) The expression of cytochrome c and Bax in the cytosolic fraction was measured by western blotting. β-Actin expression was examined as the protein loading control; (**B**) The expression of cytochrome c and Bax in the mitochondrial fraction was detected by western blotting. COX IV expression was examined as the protein loading control; (**C**) The expression of Bcl-2 and Bax were tested by western blotting. β-Actin as a loading control. Values were represented as mean ± SD (*n* = 6, each group). ^##^ *p* < 0.01 vs. normal; ** *p* < 0.01 vs. H/R-treated cells; ^$^ *p* < 0.05, ^$$^ *p* < 0.01 vs. H/R + BCF-treated cells.

To further explore the molecular mechanism of BCF inhibited H/R-induced mitochondrial dysfunction, the translocation of Bax, the release of cytochrome c, and the expression of Bcl-2 were measured by western blotting. As shown in Figure 5, compared with the normal group, Bax was increased in the mitochondria (*p* < 0.01, Figure 5B) and decreased in the cytosol (*p* < 0.01, Figure 5A) in the H/R group. On the other hand, cytochrome c was released into the cytosol as a result of Bax translocation (*p* < 0.01). However, the translocation of Bax and the release of cytochrome c were both reduced in the H/R + BCF group, accompanied by the increase of the Bcl-2/Bax expression ratio (*p* < 0.01 or *p* < 0.05 vs. H/R group, Figure 5). When co-administering with LY294002, the above effects of BCF were partially attenuated (*p* < 0.05 or *p* < 0.01).

## 3. Discussion

Myocardium I/R injury plays a key role in the development of coronary heart diseases [15]. Apoptosis contribute to one of the important pathological mechanisms of I/R injury [6]. In the present study, we found that BCF attenuated apoptosis in H9c2 cardiomyocytes in response to H/R. Furthermore, we demonstrated that BCF prevents H/R induced apoptosis of cardiomyocytes by improving mitochondrial dysfunction via PI3K/Akt signaling pathway.

Apoptosis, a form of programmed cell death, mainly includes extrinsic (death receptor) and intrinsic (mitochondria) pathways [6]. Mitochondrial dysfunction is associated with intrinsic apoptosis, which known as the mitochondrial apoptotic pathway [5]. Multiple studies have demonstrated that mitochondrial apoptotic pathway is considered crucial in myocardial apoptosis induced by H/R [4,5]. H/R is an oxidative stress response that stimulates the generation of ROS [4]. Intracellular ROS directly interacts with mitochondrial proteins and lipids, accelerating mitochondria malfunction [16,17]. In addition, the excessive mitochondrial ROS, decreased ATP supply, ΔΨm collapsing, and MPTP opening further release cytochrome c, then activate cytochrome c-mediated caspase family, finally leading to cell apoptosis [18,19]. Here, we showed that pretreatment with BCF significantly prevented excessive ROS, remarkably inhibited the collapse of ΔΨm, the reduction of mitochondrial ATP production, and the MPTP opening, and also clearly blocked the release of cytochrome c and depressed the activation of caspase-3. These results suggested that pretreatment BCF prevented H/R induced apoptosis by improving mitochondrial malfunction.

Accumulating evidence suggests that the main regulators of the mitochondrial apoptosis pathway are the Bcl-2 family proteins [20]. In healthy cells, Bax appears to exist as a monomer in the cytosol, however, when apoptosis induction, it translocates specifically to mitochondria [21,22]. The translocation of Bax causes the loss of ∆Ψm and ATP content, MPTP opening, mitochondrial swelling, and rupture of the outer mitochondrial membrane (OMM) [23,24]. The loss of ∆Ψm and rupture of OMM allow for the release of ROS and cytochrome c and subsequently induce apoptotic cell death [25]. The anti-apoptotic family members of Bcl-2 protein family, such as Bcl-2, can bind Bax and further inhibit Bax to promote cell survival [21]. In the present study, compared with H/R group, BCF pretreatment significantly increased the Bcl-2/Bax expression ratio and attenuated the translocation of Bax. These results indicated that the regulation of Bcl-2 family might be partially involved in the mechanisms of BCF on improving mitochondrial dysfunction.

PI3K/Akt signaling pathway is an important antiapoptosis/proliferation signaling pathway [7]. When PI3K is phosphate-activated by the stimulation of extracellular signal molecules, Akt conformation changes into p-Akt then affects its downstream substrates, which promotes cell proliferation and inhibits apoptosis. Our data showed that BCF pretreatment, upregulated the expression of p-PI3K (Tyr467) and p-Akt (Ser473), both in normal and H/R treated cardiomyocytes (*p* < 0.01, Figure 2). Therefore, we deduced that BCF can activate PI3K/Akt signaling pathway. On the other hand, as shown in Figure 3, Figure 4 and Figure 5, a PI3K special inhibitor, LY294002 significantly blocked the BCF-mediated activation of Akt, which led to a significant decrease in Bcl-2/Bax ratio, as well as caused mitochondrial malfunction and activation of caspase-3 (*p* < 0.05 or *p* < 0.01 vs. H/R + BCF group). Thus, we can infer that PI3K/Akt pathway plays an important role in the anti-apoptosis effect of BCF.

## 4. Materials and Methods

### 4.1. Chemicals and Materials

BCF was obtained from our lab (With rutin as a reference substance, the total flavonoid content of BCF was 82%). The extraction and isolation processes of BCF were previously presented [14].

All cell culture materials were from GIBCO (Grand Island, NY, USA). Cell Counting Kit-8 was acquired from Dojindo Molecular Technologies Inc. (Gaithersburg, MD, USA). Hoechst 33342 and LY294002 was obtained from Sigma-Aldrich Inc. (St. Louis, MO, USA). The Alexa Fluor^®^ 488 annexinV/Dead Cell Apoptosis Kit and MitoSOX Red Mitochondrial Superoxide Indicator were acquired from Invitrogen (Carlsbad, CA, USA). The JC-1, ATP assay kit, and Cell Mitochondria Isolation Kit were obtained from Beyotime Biotechnology Inc. (Beijing, China). The living cell mitochondrial permeability transition pore (MPTP) fluorescence detection kit was acquired from GENMED (Arlington, MA, USA). The primary antibodies against p-Akt, Bcl-2, Bax, caspase-3, COX IV, and Cytochrome c were from Cell Signaling Technology Inc. (Danvers, MA, USA). All other antibodies were purchased from Santa Cruz Biotechnology (Dallas, TX, USA).

### 4.2. Cell Culture and Hypoxia-Reoxygenation Model (H/R)

H9c2 cardiomyocytes (Cell Bank of the Chinese Academy of Sciences, Shanghai, China) were cultured in high glucose DMEM supplemented with 10% (*v*/*v*) fetal bovine serum, 1% penicillin/streptomycin (*v*/*v*), and 2mM L-glutamine. The cells were maintained in a humidified incubator with 95% air and 5% CO_2_ at 37 °C. The H/R model was built using a modified process [26]. Briefly, high glucose DMEM medium was changed with non-glucose DMEM to mimic ischemia. Then the H9c2 cardiomyocytes were incubated at 37 °C in an anaerobic glove box (Coy Laboratory Products Inc., Grass Lake, MI, USA), where normal air was removed by a combination of 5% CO_2_, 5% H_2_, and 90% N_2_. The H9c2 cardiomyocytes were cultured under hypoxia for 6 h and then removed to the regular incubator for 12 h with the medium replaced by high glucose medium to mimic reperfusion. The corresponding normal cells were incubated under normoxic conditions for equivalent durations with high glucose DMEM. For all experiments, cells were plated at an appropriate density according to the experimental design and grown for 24 h to reach 70%–80% confluence before experimentations began.

### 4.3. Experimental Protocols

The cultured H9c2 cardiomyocytes were randomly divided into different groups. In the normal group, H9c2 cardiomyocytes were incubated under normal air conditions for equivalent durations with high glucose DMEM. The H/R group was conducted as described in the preceding section. In the H/R + BCF group, the H9c2 cardiomyocytes subjected to H/R were pretreated with BCF for 4 h (The pretreatment method of BCF is determined according to the preliminary experiment. Figure S1, Supplementary Materials). The BCF group was treated the same as the normal group, but the cells were incubated with BCF for 4 h. The H/R + BCF + LY group was processed the same as the H/R + BCF group, but the cells were incubated with 20 μM LY294002 for 1 h before treated with BCF (The processing method of the inhibitor is determined according to the literature [27] and the CCK-8 assay. Figure S2). Three experimental categories were as follows: the first included the normal, H/R, and H/R + BCF (0.78125, 3.125 and 6.25 μg/mL) groups; the second included the normal, H/R, H/R + BCF (3.125 μg/mL), and BCF (3.125 μg/mL) groups; and the last included the normal, H/R, H/R + BCF (3.125 μg/mL), and H/R + BCF (3.125 μg/mL) + LY(20 μM) groups.

### 4.4. Cell Viability Analysis

Cell viability was determined by Cell Counting Kit-8. The cells were seeded at 1 × 10^4^ cells/well in 96-well plates. After 4 h of treatment with different concentrations of BCF (0.78125, 3.125, and 6.25 μg/mL) followed by H/R, 10 μL tetrazolium salt WST-8 (1/10 dilution) was added to each well and incubated for 3 h. In the cytotoxic test, cells were treated with different concentrations of BCF for 24 h before incubated with tetrazolium salt WST-8. The absorbance was determined by a microplate reader (Tecan Austria GmbH., Grödig, Austria) at 450 nm.

### 4.5. Flow Cytometric Detection of Apoptosis

The percentages of early apoptosis and necrosis were measured using an Alexa Fluor^®^ 488 annexin V/Dead Cell Apoptosis Kit for flow cytometry according to the manufacturer’s instructions (Invitrogen). After treatment, the cells were harvested and washed twice with cold PBS, and then incubated with 5 μL FITC-Annexin V and 1 μL PI working solution (100 μg/mL) for 15 min in the dark at room temperature. Cellular fluorescence was measured by flow cytometry analysis (FACS CaliburTM, BD Biosciences, San Jose, CA, USA).

### 4.6. Measurement of ROS-Generating Mitochondria

Mitochondria-associated ROS levels were measured by staining cells with MitoSOX according to the manufacturer’s protocol. Briefly, after treatment, the H9c2 cells were incubated with MitoSOX Red reagent (5 μM) for 10 min at 37 °C. Then the cells were incubated with Hoechst 33342 (10 μg/mL) in the dark for 15 min at 37 °C. After staining, cells were washed three times with warm PBS and then observed under a fluorescence microscope (EVOS^®^ FL Imaging System, Thermo Fisher Scientific, Carlsbad, CA, USA). Besides, the florescence intensity of the cells was determined on a microplate reader (Tecan Austria GmbH.) at an excitation wavelength of 510 nm and an emission wavelength of 580 nm.

### 4.7. Determination of Mitochondrial Transmembrane Potential (∆Ψm) and ATP Production

We used 5, 50, 6, 60-tetrachloro-1, 10, 3, 30-tetraethylbenzimidazolyl-carbocyanine iodide (JC-1) to analyze changes in the mitochondrial transmembrane potential. Following drug treatment, the cells were incubated with JC-1 (10 μg/mL) for 30 min at 37 °C in the dark. Then, the cells were washed twice with PBS and were monitored immediately with fluorescence microscope. Besides, the florescence intensity of the cells was determined on a microplate reader at excitation wavelength of 514 nm and the emission wavelength of 529 nm. The aggregates were detected at an excitation wavelength of 585 nm and an emission wavelength of 590 nm.

The ATP production assay was performed according to the kit instructions. After centrifugation to remove cell debris, the supernatant was added to the substrate solution. The luminescence was recorded in a microplate reader (Tecan Austria GmbH.) with an integration time of 10 s per well.

### 4.8. Determination of Changes in the MPTP

The MPTP opening in H9c2 cardiomyocytes was measured by the living cell MPTP fluorescence detection kit. Following drug treatment, the cells were washed twice with the reagent A, and then incubated with intermixture of reagent B and reagent C for 20 min at 37 °C in the dark. Next, the cells were incubated with Hoechst 33342 (10 μg/mL) in the dark for 15 min. After staining, cells were washed twice with reagent A followed by image acquisition using fluorescence microscopy. Besides, the florescence intensity of the cells was determined by a microplate reader at excitation wavelength of 488 nm and the emission wavelength of 505 nm.

### 4.9. Western Blotting Analysis

Cells were harvested and protein samples were extracted as described [10]. The lysates of cytoplasm and mitochondria were prepared by Cell Mitochondria Isolation Kit (Beyotime Biotechnology Inc., Beijing, China) according to the manufacturer’s protocol. Equal amounts of the protein (30 μg) from each sample were separated by SDS-PAGE and transferred onto nitrocellulose membranes. After blocking with 5% non-fat dry milk powder in Tris-buffered saline containing 0.1% Tween-20 (TBST) for 4 h, the membranes were incubated overnight at 4 °C with the following primary antibodies: PI3K (p85α, 1:200), p-PI3K (Tyr467, 1:200), Akt (1:200) p-Akt (Ser473, 1:1000), caspase-3 (1:1000), Bcl-2 (1:1000), Bax (1:1000), COX IV (1:1000), Cytochrome c (1:1000). Then, the membranes were washed with TBST and incubated for 1.5 h with peroxidase-conjugated IgG (1:2000) at room temperature. Finally, the membranes were washed in TBST and developed using an ECL chemiluminescence detection system.

### 4.10. Statistical Analyses

Each sample was assayed in triplicate. The results were expressed as means ± standard deviation. Comparisons between different groups were performed using one-way ANOVA followed by post hoc analysis with a Student–Newman–Keuls test. Statistical significance was considered at *p* < 0.05.

## 5. Conclusions

**Figure 6:**
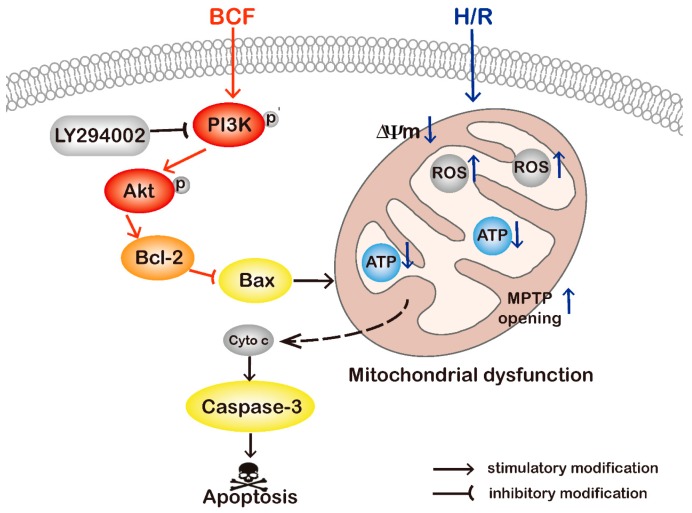
Schematic diagram of the mechanism of BCF alleviates H/R induced apoptosis in H9c2 cells by improving mitochondrial dysfunction through activating the PI3K/Akt pathway.

In summary, this study indicated for the first time that BCF alleviated H/R induced apoptosis in H9c2 cells by improving mitochondrial dysfunction, at least in part, through activating PI3K/Akt pathway. It might provide some references for the development of BCF into cardiovascular drugs. However, more advanced research is necessary to further explore the mechanisms of BCF against I/R–induced myocardial damage (Figure 6).

## Supplementary Materials

Supplementary materials can be accessed at: http://www.mdpi.com/1420-3049/21/11/1469/s1. Figure S1. Effects of BCF on H9c2 cell viability; Figure S2. Effects of LY294002 on H9c2 cell viability.
