# Supplementary material for: Bauhinia championii Flavone Attenuates Hypoxia-Reoxygenation Induced Apoptosis in H9c2 Cardiomyocytes by Improving Mitochondrial Dysfunction
Source: Molecules. 2016 Nov 4;21(11):1469. doi: 10.3390/molecules21111469 (PMC6273835; doi:10.3390/molecules21111469)
Supplement: Supplementary file 1 [file molecules-21-01469-s001.pdf]

Supplementary Materials: *Bauhinia championii* Flavone Attenuates Hypoxia-Reoxygenation Induced Apoptosis in H9c2 Cardiomyocytes by Improving Mitochondrial Dysfunction

Ping Liao, Guibo Sun, Chan Zhang, Min Wang, Yao Sun, Yuehan Zhou, Xiaobo Sun and Jie Jian

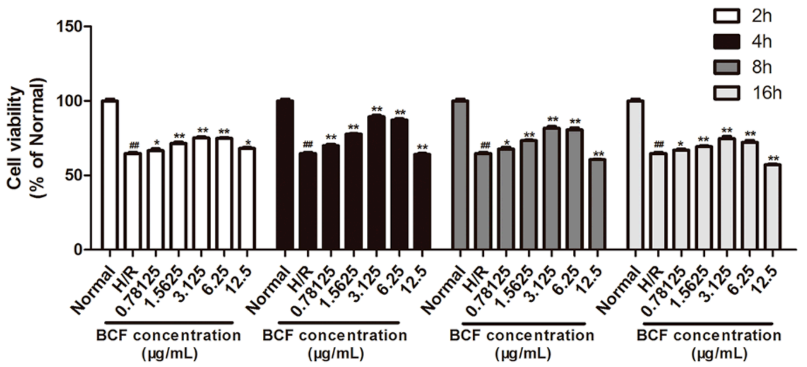

Figure S1. Effects of BCF on H9c2 cell viability. H9c2 cells were pretreated with the indicated BCF concentrations for 2 h, 4 h, 8 h or 16 h followed by H/R. Cell viability was determined by CCK-8 assay. Values represent mean ± SD (n = 6, each group). ## $p < 0.01$ vs. normal; * $p < 0.05$, ** $p < 0.01$ vs. H/R-treated cells.

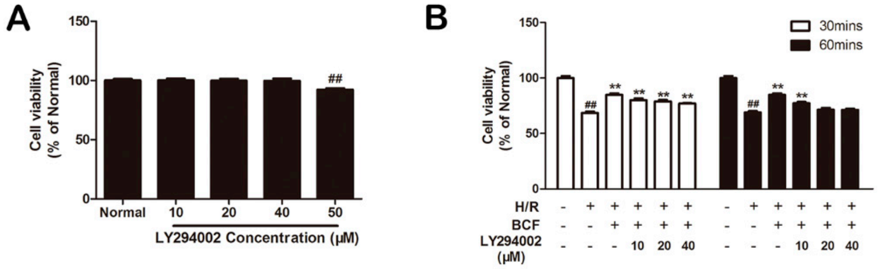

Figure S2. Effects of LY294002 on H9c2 cell viability. (**A**) H9c2 cells were pretreated with the varying concentrations of LY294002 for 1 h. Cell viability was determined by CCK-8 assay; (**B**) H9c2 cells were pre-incubated with indicated LY294002 concentrations for 30min or 60min, and then pretreated with BCF (3.125μg/mL) for 4 h followed by H/R. Cell viability was determined by CCK-8 assay. Values represent mean ± SD (n = 6, each group). ## $p < 0.01$ vs. normal; ** $p < 0.01$ vs. H/R-treated cells.

1. The Extraction and Isolation Processes of BCF

The powder of dried plant of *Bauhinia championii (Benth.) Benth.* (10 kg) was extracted with 78 L 70% ethanol for 3 times, 72 h each time. All the extracts were combined and condensed under reduced pressure to afford ethanol extract (750 g). Part of the ethanol extract was suspended in water and then successively partitioned with petroleum ether (32 g), ethyl acetate (400 g) and *n*-butyl alcohol (146 g) fractions, respectively. The ethyl acetate layer was concentrated and dried in vaccum to obtain a dry residue (100 g). This dry residue was fractionated by column chromatography (CC) using a silica gel H-packed column (10 cm × 120 cm) with a gradient mixture of chloroform and methyl alcohol (0%–100% methyl alcohol, 2500 mL each fraction). Then, all fractions were combined according to thinlayer chromatography

(TLC) analysis and afforded 8 fractions (Fr. **1–8**). The antioxidative activities of Fr. **1–8** were evaluated as previously reported [1] for further study. Among these 8 fractions, the antioxidative activity of Fr .**2** was the highest. With rutin as a reference substance, the total flavonoid content of Fr. **2** (i.e., *Bauhinia championii* flavones [BCF]) was 82%.

So Fr .**2** (2.3 g) was further fractioned by CC (Agil*ent* ZORBAX SB-C18, 250 mm × 10 mm, 5 μm) to obtain the eight sub-fractions as follows: Fr. **2-1**(18.5 mg): moving phase: 20% methyl alcohol at 3 mL/min; Fr. **2-2** (4.8 mg): moving phase: 23% methyl alcohol at 3 mL/min; Fr. **2-3** (4.2 mg) and Fr. **2-8** (6.6 mg): moving phase: 18% methyl alcohol at 3 mL/min; Fr. **2-4** (25.2 mg): moving phase: 20% methyl alcohol at 3mL/min; Fr. **2-5** (4.1 mg) and Fr. **2-6** (4.7 mg): moving phase: 23% methyl alcohol at 3 mL/min; Fr. **2-7** (6.3 mg): moving phase: 16% methyl alcohol at 3 mL/min.

Eight chemical constituents were separated from BCF for the first time as follows: catechin (**1**), (−)-epicatechin (**2**), catechin-3-*O*-α-L-rhamnopyranoside (**3**), (−)-epigallo-catechin-3-*O*-gallate (**4**), dimethyl(R)-hexahydroxydiphenoate (**5**), 4-hydroxy-3-methoxy-phenyl-1-*O*-(6′-*O*-galloyl)-β-D-glucopyranoside (**6**), 3,5-dimethoxy-4-hydroxyphenol-1-*O*-β-D-(6′-*O*-galloyl)-glucopyranoside (**7**), rocymosin A (3,4-dihydroxyphenethyl alcohol 4-*O*-D-(6″-*O*-galloyl)-glucopyranoside) (**8**).

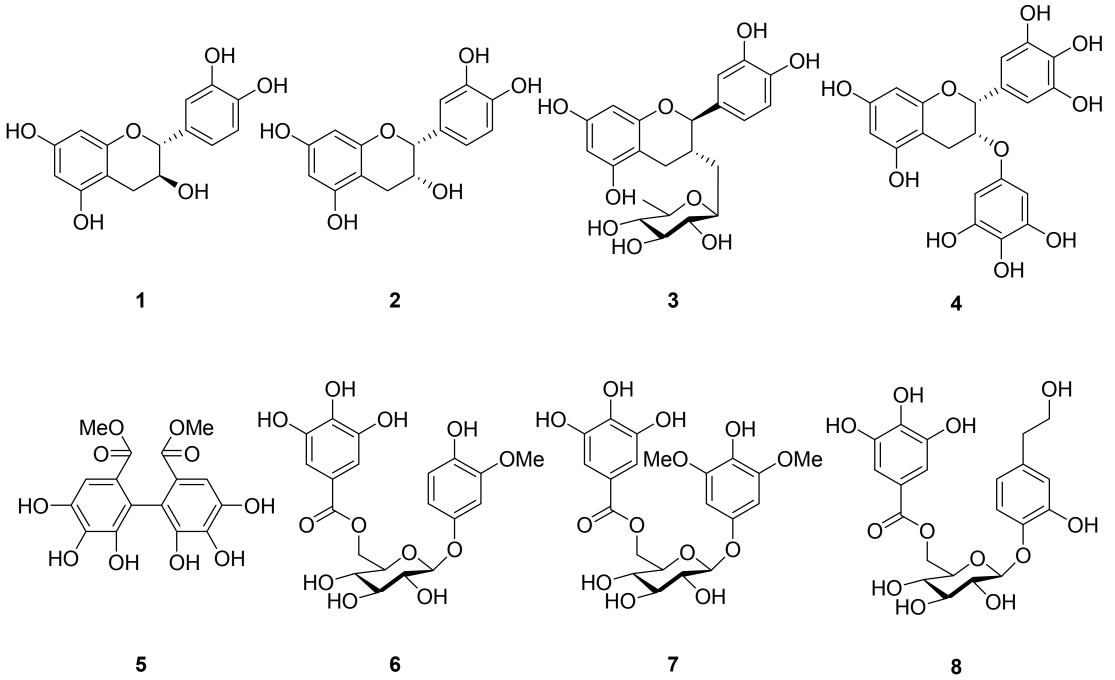

Figure S3. The chemical structure of BCF constituents.

References

1. Emam, A.M.; Mohamed, M.A.; Diab, N.Y.; Megally, N.Y. Isolation and structure elucidation of antioxidant compounds from leaves of *Laurus nobilis* and *Emex spinosus*. *Drug Discov. Ther.* **2010**, *4*, 202–207.
